# Dysmorphic Short Stature: Radiological Diagnosis of Trichorhinophalangeal Syndrome

**DOI:** 10.1155/2018/5189062

**Published:** 2018-11-21

**Authors:** Corina Ramona Nicolescu, Laura Kasongo, Léon Rausin

**Affiliations:** ^1^Division of Endocrinology and Diabetes, Department of Pediatrics, University of Liege, Centre Hospitalier Regional de la Citadelle, Bld du 12eme de Ligne 1, 4000 Liege, Belgium; ^2^Radiology Department, University of Liege, Centre Hospitalier Regional de la Citadelle, Bld du 12eme de Ligne 1, 4000 Liege, Belgium

## Abstract

Trichorhinophalangeal syndrome (TRPS), a type of skeletal dysplasia, is characterized by a triad of dysmorphic (bulbous nose and large ears); ectodermal (thin and sparse hair); and skeletal (short stature and cone-shaped epiphyses) findings, and this combination is helpful for early diagnosis and appropriate follow-up. A 14-year-old boy presented with short stature and distinctive facial features, and following the first clinical and biological evaluation, no precise diagnosis was reached. Progressive bilateral development of noninflammatory and painless deformity of his second finger required a radiological exam that highlighted the key elements (cone-shaped epiphyses) for final diagnosis. This case illustrates the difficulties to early recognition of TRPS when the clinical presentation is not complete and radiological findings are missing.

## 1. Introduction

Skeletal dysplasias are a group of 436 well-delineated disorders [1], caused by disturbance of bone growth and characterized by variable phenotypical expressions, including short stature (typically nonendocrine), facial deformities, multisystem involvement (ocular, cutaneous, cardiac, gastrointestinal, genital, and neurological), and different radiological abnormalities. The genetic background becomes more and more detailed, with 364 genes described today [1].

Trichorhinophalangeal syndrome (TRPS) is a rare inherited skeletal dysplasia with characteristic physical features (pear-shaped nose, thin and sparse hair, and prominent ears) and abnormalities of the fingers and/or toes. As the range and severity of the phenotype expression is variable, the clinical diagnosis can be missed or unreported. The diagnosis is usually suspected on a physical background and confirmed by radiographic studies of the skeleton, showing distinctive abnormalities of the hands, feet (epiphyseal coning), and pelvis, (small, flat, or fragmented femoral heads in some patients). Molecular genetic analysis can be used to identify mutations of the TRPS gene.

Three different types are described (types I, II, and III) with some common and distinctive clinical and radiological features and different genetic abnormalities.

## 2. Case Presentation

A 14-year-old adolescent boy was referred to our Endocrinology Department for evaluation of short stature. As no medical records were available, previous growth velocity could not be evaluated. The patient reported that he had always been short for his age throughout his childhood.

His recent medical history was negative for headaches, vomiting, or vision changes. There were no reports of fatigue, cold intolerance, constipation, and skin or hair changes. Appetite was normal with no recent weight loss. There were no academic concerns. He regularly played football, with no history of traumatic or nontraumatic fractures. He took no medications. For several months, he had reported minor bilateral symmetrical crookedness on his second fingers, without any pain or local symptoms.

He had been born at 36 gestational weeks, weighing 2450 g with no history of abnormal gestation, breech presentation, ischemic insult at birth, or other neonatal events.

Parental heights were normal, with a target height of 177.5 cm. Parental pubertal timing was also within normal limits. Family history was negative for short stature, endocrine, or autoimmune conditions.

**Figure 1:**
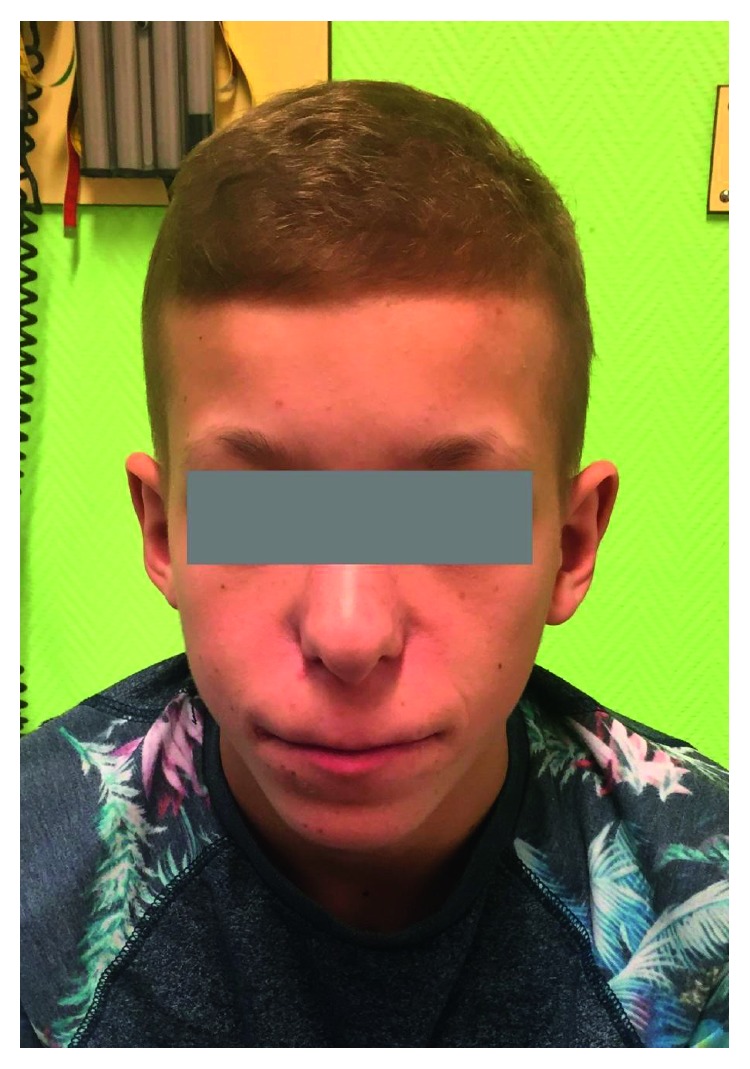
Typical dysmorphic facial features.

Clinical examination revealed a dysmorphic, proportionate, and relatively short adolescent with normal vital signs. His anthropometric parameters were −2 SDs (standard deviations) for weight (37 kg) and between −2 SDs and −2.5 SDs for height (148 cm) (Belgian charts). His height was below his midparental genetic interval. His upper:lower segment ratio and arm span were normal. His hands and feet appeared short, with middle, painless tumefaction of soft tissue around the index proximal interphalangeal joints. No spinal abnormalities were noted, but a mild pectus excavatum was present. There was no thyromegaly. Testicular and pubic hair development was in Tanner stage II (testicular volume 6 ml). The most prominent dysmorphic features were a pear-shaped nose, a thin upper lip with small lower jaw, prominent ears, and markedly thin and sparse blonde hair with rarefaction of the lateral eyebrows (Figure 1). The teeth examination was difficult because he wore dental braces to correct dental irregularities.

An extensive biological workup was performed. Complete blood count and serum levels of inflammatory markers, electrolytes, glucose, renal function, liver enzyme, and tissue transglutaminase antibody levels were normal. The laboratory findings showed normal thyroid and adrenal function and normal growth hormone secretion (normal levels of insulin-like growth factor and insulin-like growth factor binding protein). Additional pituitary and gonad testing revealed a normal prolactin level and pubertal testosterone, luteinizing hormone (LH), and follicle-stimulating hormone (FSH) levels (Table 1). The bone age was evaluated at 12 years 6 months.

A renal and cardiac ultrasound looking for other possible somatic malformations [2] wasperformed and showed normal morphological kidneys, ureters, and urinary bladder and no cardiac abnormalities.

After this first clinical, biological, and radiological evaluation, the working diagnosis was constitutional growth delay with late normal puberty, but skeletal dysplasia (dysmorphic features in the context of short stature) was considered for differential diagnosis.

During the follow-up 6 months later, the initial apparent soft tissue tumefaction of his indexes had progressed to localized noninflammatory and painless deformity, causing limited difficulty in writing and typing on a keyboard. This sign was isolated, and the patient did not complain of pain or history of local trauma or infection. At this time, his clinical appearance was normal, except for the aforementioned dysmorphic traits. His growth velocity (5 cm/6 months) was normal in the context of spontaneous pubertal progress (Tanner stage III, testicular volume 8–10 ml).

**Figure 2:**
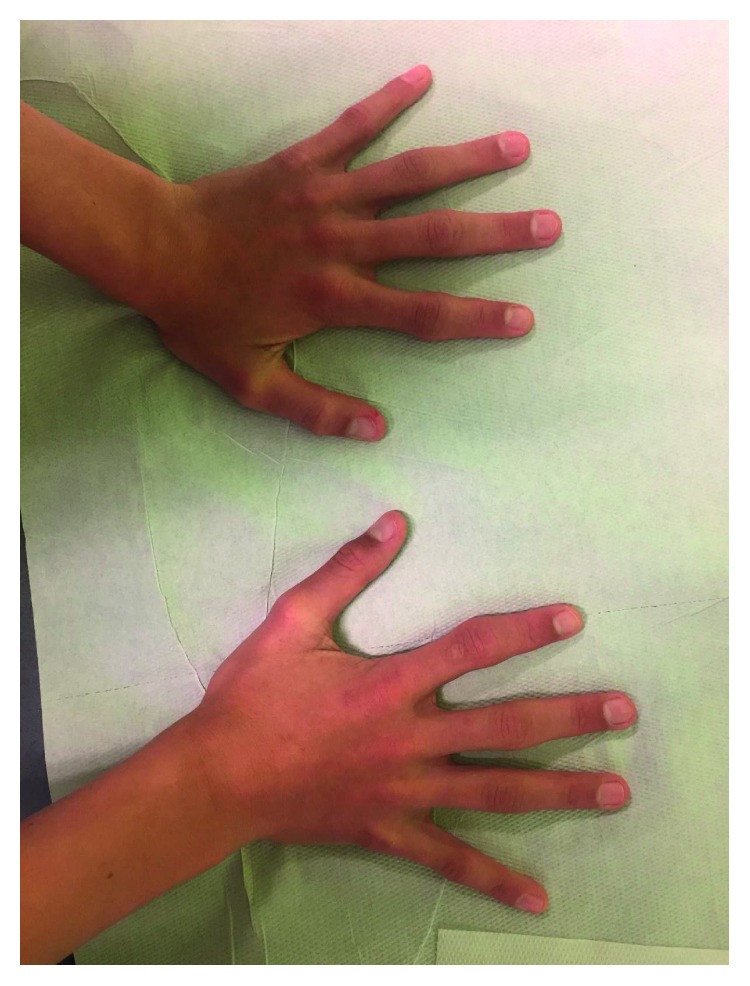
Symmetrical deformity of proximal interphalangeal joints of both hands.

Clinical examination of his hands revealed isolated ulnar deviation of the second fingers and symmetrical deformity of proximal interphalangeal joints of both hands (Figure 2). He had no other painful or deformed joints.

**Figure 3:**
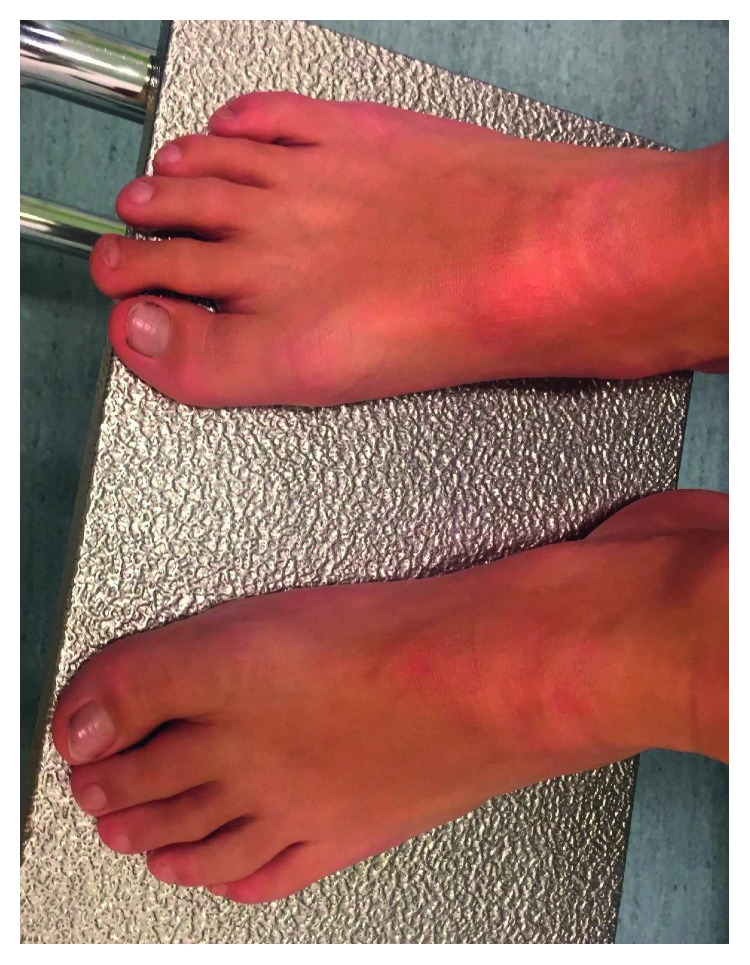
Feet abnormalities with short toes.

His feet look normal, but the toes are short (Figure 3).

Theoretically, a differential diagnosis with inflammatory arthritis was considered, but the clinical findings (dysmorphic features, short stature, and relatively asymptomatic finger abnormalities) were more suggestive of a skeletal dysplasia. Radiographs of hands, feet, and pelvis were performed and allowed the definitive diagnosis.

**Figure 4:**
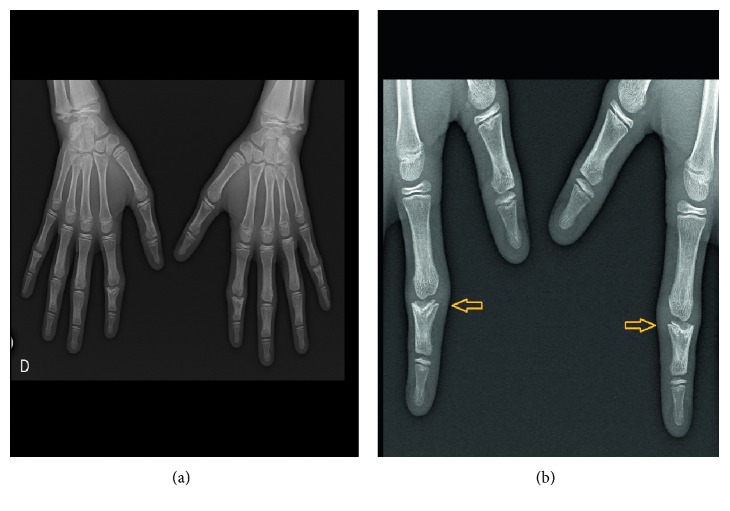
(a) Cone-shaped epiphyses of the middle phalanges of the second digit of both hands and (b) magnification of the cone-shaped epiphyses.

The plain radiograph of his wrists showed cone-shaped epiphyses of the middle phalanges of the second digit of both hands with moderate deviation of the phalangeal axis (Figures 4(a) and 4(b)).

**Figure 5:**
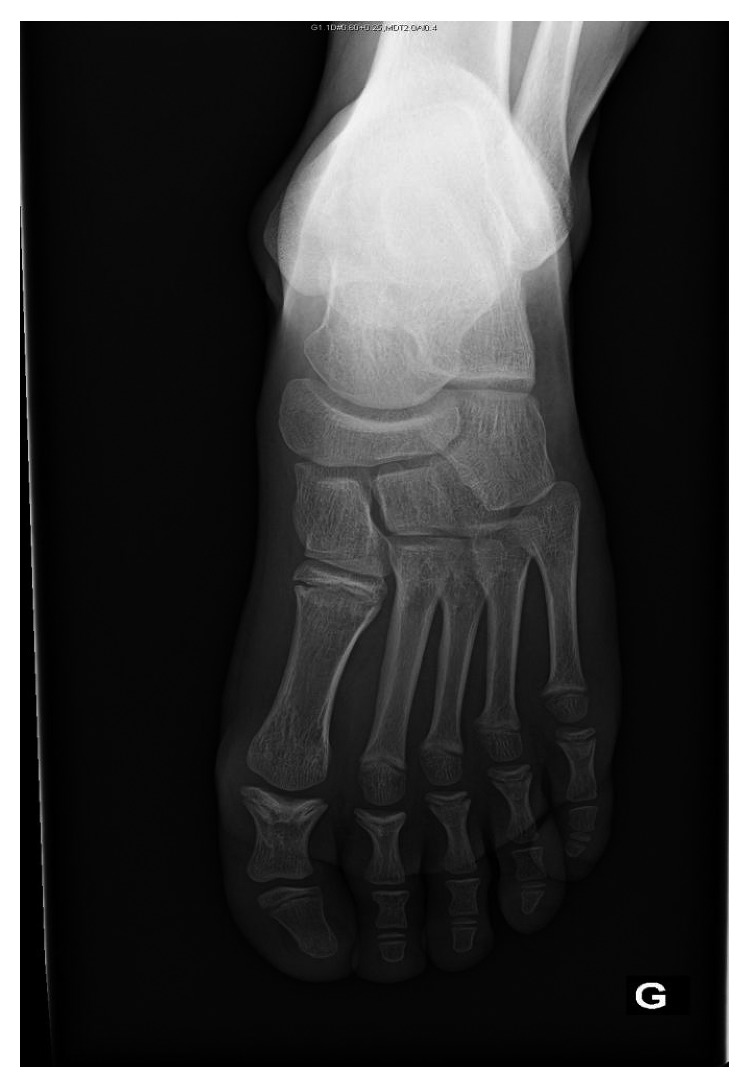
Cone-shaped epiphyses with shortness of the toes.

Similar cone-shaped epiphyses were found in the proximal phalanx of the great toe and up to the fourth one of both feet with shortness of all toes (Figure 5).

No other radiological joint impairments (juxtaarticular osteopenia or erosions) were found. Radiographs of the pelvis and whole-body magnetic resonance imaging (looking for fine abnormalities, particularly long bone cysts not visible on plain radiographs) [3] were normal. The bone age was retarded at 12 years 6 months (the chronological age was 14 years 6 months).

Taking together the clinical and particularly the radiological findings, the correct diagnosis of trichorhinophalangeal syndrome (TRPS) was achieved. This was a delayed diagnosis, and our explanations for this include the underrecognition of the dysmorphic features (in our first examination), the underinterpretation of fingers tumefaction in the clinical context, and consecutively no hand radiological exam being conducted.

Other entities associating ectodermal and skeletal phenotypes were reviewed, particularly Albright osteodystrophy, acrodysostosis, and other brachydactyly syndromes, but several features of the presentation were inconsistent with these diagnoses.

Cytogenetic analysis was not performed. The family history appeared to be negative, his parents and his sister do not manifest the syndrome phenotype, and in such conditions, a sporadic case of TRPS type I was considered highly possible.

## 3. Discussion

TRPS is a rare skeletal disease associated with craniofacial, ectodermal, and skeletal findings. Two types are described with relatively similar clinical phenotypes but with distinctive radiological and genetic characteristics.

Defined by Giedion in 1966, type I is characterized by a triad of craniofacial, ectodermal, and skeletal abnormalities, with high penetrance and variable expressivity.

The most dysmorphic facial trait concerns the large nose, with a broad ridge and tip and underdeveloped alae. Other facial findings include scanty lateral eyebrows, a long philtrum with thin upper vermillion, maxillary prognathism with mandibular hypoplasia, and large prominent ears. The ectodermal elements include fine, sparse, depigmented, and slow-growing hair and dystrophic nails. The skeletal characteristics specifically concern the hands and feet, with a clinical appearance of brachydactyly with ulnar or radial deviation of the fingers and short feet. The most characteristic radiologic abnormality involves the middle phalanges of the hands and feet and consists of an enlarged, irregular metaphyseal ending, with the shape of a cone or inverted V. The proximal or distal phalanges and some tubular bones (metacarpals) could also be affected [4]. Hip dysplasia (coxa plana, coxa magna, and coxa vara) is frequently found [5], and in pediatric patients, this hip involvement requires particular attention in order to provide a differential diagnosis with Perthes disease.

Epiphyseal changes are associated with retarded skeletal age and short stature, with a final adult height below the 50^th^ percentile [6]. Other skeletal abnormalities, such as severe osteoporosis, have also recently been reported [7]. The diagnosis of TRPS I is clinical and radiological due to the association of characteristic physical features and specific abnormalities of the hands and feet, particularly epiphyseal coning. Molecular genetic analysis can reveal a heterozygous pathogenic variant in the *TRPS1* gene on chromosome 8 (deletions, nonsense, and missense mutations). There are no genotype-phenotype correlations in TRPS I, and a certain clinical variability (sometimes with subtle clinical changes) is observed within and among families with the same *TRPS1* pathogenic variant [5, 8, 9].

Many cases could remain undiagnosed until one or several disturbing signs (short stature, sparse hair, painless swelling of the proximal interphalangeal joints, and hip pain) bring the patient to seek medical attention.

TRPS type II (Langer–Giedion syndrome) presents with the same phenotype as TRPS type I, and its characteristic features include multiple osteochondromas (scapulae, elbows, and knees) and mild-to-moderate intellectual disability [10]. Bone involvement also includes cone-shaped epiphyses of the phalanges of the hands with deformity of the fingers, delayed bone age, osteochondromas, and hip malformations. Multiple long bone cysts with pathological fractures have recently been described in a case report [3].

Most individuals with TRPS II have de novo contiguous gene deletion involving the TRPS1, RAD21, and EXT1 on chromosome 8.

TRPS type III (Sugio-Kajii syndrome) is an extremely rare autosomal dominant variant presenting a similar dysmorphic phenotype with abnormalities of the hands and feet. Inflammation of the bone and cartilage in the spinal column (osteochondritis) and spinal abnormalities (scoliosis) is relatively particular for this type.

The endocrine elements of TRPS, particularly delayed bone age during childhood and decelerated growth with adult short stature, are well described clinically, but their underlying pathological mechanisms remain poorly understood.

The currently available clinical therapies for patients with skeletal dysplasia, including the TRPS, are predominantly palliative in nature. Joint pain can be alleviated using nonsteroidal antiinflammatory drugs and the progression of skeletal deformations/abnormalities can be diminished by physiotherapy, occupational therapy, or in extreme cases, by prosthetic hip implantation (severe hip dysplasia).

Reduced linear growth and subsequently adult short stature are usually found in TRPS syndrome. The growth hormone secretion is usually normal, but when the height velocity is abnormal for pubertal development and sex, the diagnosis of growth hormone deficiency should be evaluated. Several TRPS cases with growth hormone deficiency or insensitivity are reported [11, 12]. Growth hormone therapy has been shown to improve growth and height outcome in TRPS children, but to date the definitive results are still missing.

Several pharmacotherapeutic options/therapies are under investigation for certain skeletal dysplasias, and these include in utero stem cell transplantation, stem cell transplantation, chaperone therapy, gene therapy, bone marrow transplant, C-type natriuretic peptides, and enzyme replacement therapy [13].

## 4. Conclusion

We reported a case of a short adolescent with clinical and radiological features that were highly suggestive for TRPS type I. The first clinical diagnosis (constitutional growth delay versus skeletal dysplasia) was incorrect, but 6 months later, collaborative work with the radiology team considerably narrowed the diagnosis options to those among the skeletal dysplasia types. Interpreting the clinical phenotype and the radiological aspects, namely, cone-shaped epiphyses, together was very helpful to the diagnostic approach. A confirmatory genetic diagnosis was not available.

## Figures and Tables

**Table 1:** Patient's endocrine workup.

	Patient	References
Thyroid function		
TSH	2.7	0.5–4.5 mU/L
T4	10.25	4.5–12.5 *µ*g/dL
Growth hormone secretion		
IGF1	345	106–435 ng/mL (Tanner stage II)
IGFBP3	5.0	2.8–6.3 *µ*g/mL (Tanner stage II)
Adrenal function		
Cortisol	530	150–800 nmol/L
ACTH	54	8–90 ng/L
Prolactin	5	2–18 ng/mL
Gonadotrophins		
FSH	5.0	4.7–9.5 mIU/mL
LH	4.3	2.1–13 mIU/mL
Gonadal function Testosterone	1200	310–7200 ng/L

IGF1, insulin-like growth factor 1; IGFBP3, insulin-like growth factor binding protein 3; ACTH, adrenocorticotropic hormone.
